# Severe gastric variceal haemorrhage due to splenic artery thrombosis and consecutive arterial bypass

**DOI:** 10.1186/1471-2482-11-14

**Published:** 2011-06-28

**Authors:** Klaus T von Trotha, Marcel Binnebösel, Son Truong, Florian F Behrendt, Hermann E Wasmuth, Ulf P Neumann, Marc Jansen

**Affiliations:** 1 Department of Surgery, University Hospital of the RWTH Aachen, Germany; 2 Department of Diagnostic Radiology, University Hospital of the RWTH Aachen, Germany; 3 Department of Medicine III, University Hospital of the RWTH Aachen, Germany; 4 Department of Surgery, HELIOS Hospital Emil von Behring Berlin, Germany

**Keywords:** Splenic artery thrombosis, upper gastrointestinal bleeding, laparoscopy, splenectomy, duplex ultra sound

## Abstract

**Background:**

Upper gastrointestinal haemorrhage is mainly caused by ulcers. Gastric varicosis due to portal hypertension can also be held responsible for upper gastrointestinal bleeding. Portal hypertension causes the development of a collateral circulation from the portal to the caval venous system resulting in development of oesophageal and gastric fundus varices. Those may also be held responsible for upper gastrointestinal haemorrhage.

**Case presentation:**

In this study, we describe the case of a 69-year-old male with recurrent severe upper gastrointestinal bleeding caused by arterial submucosal collaterals due to idiopathic splenic artery thrombosis. The diagnosis was secured using endoscopic duplex ultrasound and angiography. The patient was successfully treated with a laparoscopic splenectomy and complete dissection of the short gastric arteries, resulting in the collapse of the submucosal arteries in the gastric wall. Follow-up gastroscopy was performed on the 12^th ^postoperative week and showed no signs of bleeding and a significant reduction in the arterial blood flow within the gastric wall. Subsequent follow-up after 6 months also showed no further gastrointestinal bleeding as well as subjective good quality of life for the patient.

**Conclusion:**

Submucosal arterial collaterals must be excluded by endosonography via endoscopy in case of recurrent upper gastrointestinal bleeding. Laparoscopic splenectomy provides adequate treatment in preventing any recurrent bleeding, if gastric arterial collaterals are caused by splenic artery thrombosis.

## Background

Among the most common causes of upper gastrointestinal bleeding are ulcers of the duodenum or stomach, accounting for approximately 50% of all cases. These are followed by erosions of the duodenum or stomach, varicosis of the oesophagus or gastric fundus, and reflux disease [1]. It has been shown that 4/5 of all ulcers are located on the lesser curvature of the stomach, while more atypical localisations include corpus, fundus, and greater curvature of the stomach.

Gastric varicosis is often the result of portal hypertension in the liver. Such portal hypertension is caused by simultaneous increases in the portal vascular territory, the so-called "backward flow", as well as increased arterial blood flow within the splanchnic vascular territory, the "forward flow". This results in the development of a collateral circulation from the portal to the caval venous system. It is these portal gastric oesophageal collaterals that cause the oesophageal and gastric fundus varices. Indeed, up to 60% of all patients with liver cirrhosis experience bleeding episodes from oesophageal or corpus-/fundus varicosis bleeding [1].

In this study, we present a rare case of severe upper gastrointestinal haemorrhage caused by arterial collaterals to the spleen after thrombosis of the splenic artery. Symptoms, diagnosis, and treatment, including follow-up, are presented.

## Case presentation

**Figure 1:**
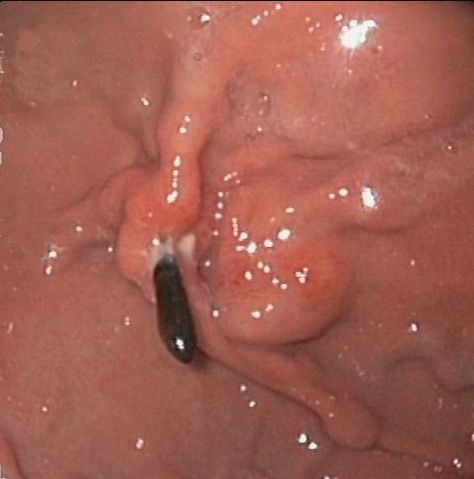
**The upper gastrointestinal endoscopy showed preoperative thick submucous varices along a small ulcer, one clip in situ**.

A 69-year-old patient was admitted for care due to recurrent upper gastrointestinal bleeding. The patient had previously received treated for similar symptoms two years prior at another facility. Upper gastrointestinal endoscopy revealed bleeding next to a varicose shaped submucosal vessel located at the fundus of the stomach (Figure 1). The bleeding could be successfully stilled with an injection of N-butyl-2-cyanoacrylate/Lipiodol and subsequent clipping.

**Figure 2:**
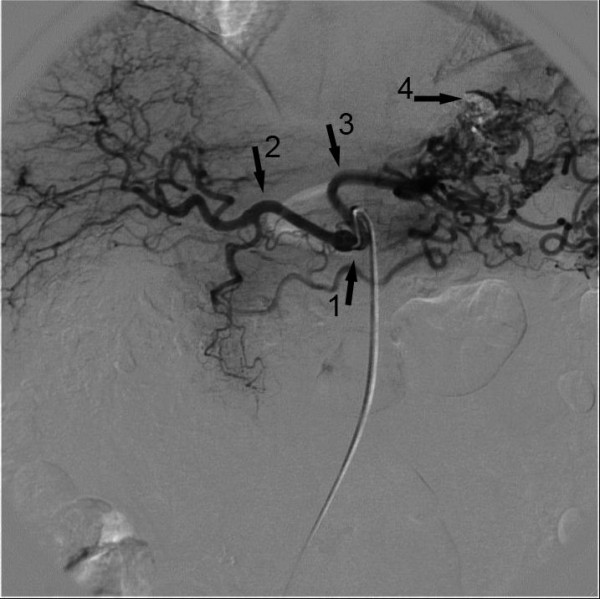
**Angiography of the the coeliac trunc (1) showing the common hepatic artery (2) and the left gastric artery with multiple collateral vessels (3)**. The splenic artery as third branch is missing. Residual of N-butyl-2-cyanoacrylate lipiodol injection (4) can be seen.

**Figure 3:**
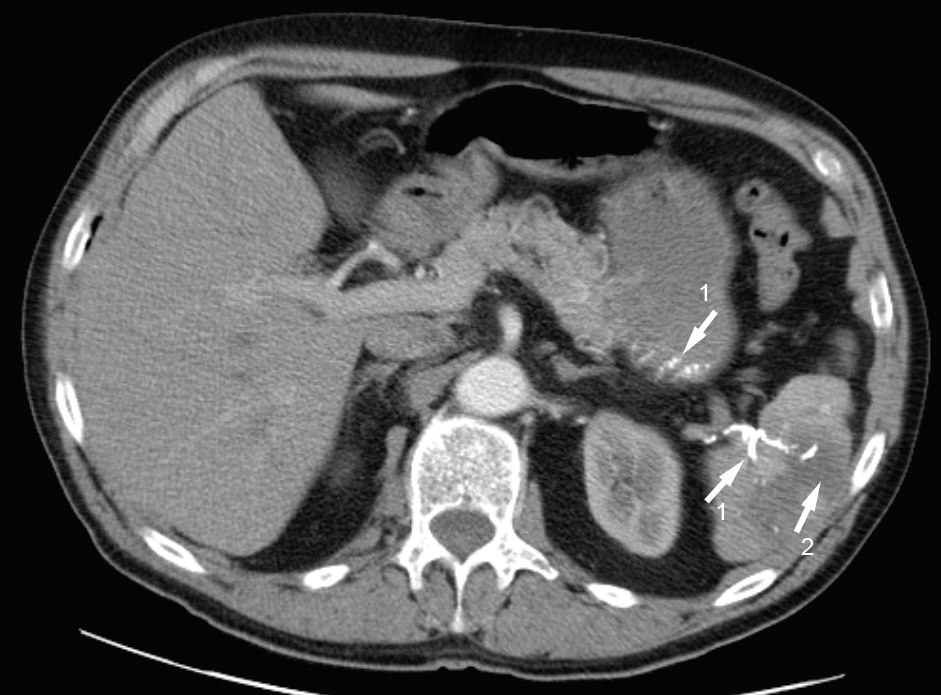
**Axial contrast enhanced CT scan of the upper abdomen showing lipiodol in the arterial collaterals of the stomach running to the spleen (1)**. Furthermore, CT revealed an infarction of splenic tissue adjacent to these vessels (2).

Further investigation, using computed tomography (CT) and conventional angiography revealed the cause of the prominent varicose shaped vessels, namely thrombosis of the splenic artery (Figure 2). Surprisingly, conventional angiography of the celiac trunk was able to demonstrate collateral vessels via the left gastric artery to the spleen. Furthermore, selective angiography of the superior mesenteric artery showed collateral vessels to the caudal portion of the spleen. In order to verify the nature of these unexpected arterial collaterals via the submucosal vessels at the gastric fundus and the reversed blood flow, a CT scan was employed, and revealed the presence of Lipiodol within the arterial collaterals as well as in the vessels of the upper portion of the spleen. Lipiodol had been injected in a mixture with N-butyl- 2-cyanoacrylate during the treatment of the gastrointestinal bleeding (Figure 3). As a complication of the therapy, the adjacent splenic tissue demonstrated signs of infarction, most likely caused by the N-butyl- 2-cyanoacrylate.

Computed tomography also showed a slight increase in liver vascularisation as indicative of possible cirrhotic remodelling. To eliminate the possibility of cirrhotic liver remodelling, a fibroscan was conducted to measure liver stiffness. The result yielded a mean score of 4.2 kPa, and thereby excluded the possibility of significant fibrosis. Also of importance was the fact there were no signs of oesophageal or gastric venous varicosis. Abdominal ultrasound was also able to safely exclude any signs of liver cirrhosis, including ascites or splenomegaly.

**Figure 4:**
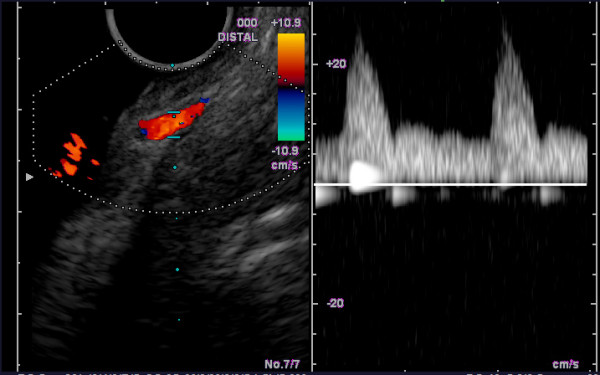
**Endoscopic colour duplex sonography showing arterial flow on the apparently gastric varices**.

In order to prove that the submucosal vessels were indeed arterial collaterals, a gastrointestinal endoscopy of the stomach using color-duplex ultrasound was performed. Multiple arterial vessels, but no venous collaterals, could be identified in the gastric fundus (Figure 4).

The examination results, in combination with a medical history of splenic artery thrombosis, suggested that the varicose shaped vessels did not originate from cirrhosis, but were in fact symptomatic collaterals caused by the thrombosis.

Pancreatitis and blunt abdominal trauma are among the known causes of splenic artery thrombosis [2,3]. However, both could be excluded given the patient's medical history. Additionally, a cardiovascular origin could be ruled out. In order to further elucidate its origin, thrombophilia diagnostic was performed. Here, no relevant anomalies were detected (Protein C (function: 107% (n: 70-149%), Protein S function: 72% (n: 75-130%), Protein S (quant.) 86% (n: 75-140%), Protein S (quant.) 97% (n: 72-150%), APC-Resistance 2.4 (n: > 2.0)). Analysis of specific clotting factors showed that factor XIII was within the normal range (80% n: 70-140%), while factor VIII was slightly elevated at x > 164% (n: 70-140%). Testing for factor V Leiden thrombophilia as well as the prothrombin G20210A-Mutation also proved unremarkable.

In order to reduce the arterial blood flow via the gastric collaterals, a laparoscopic splenectomy was performed to prevent further bleeding. Two weeks prior to the operation a triple vaccination (pneumococcus, mengingococcus, h. influenzae) was administered. No technical difficulties occurred, and the entire surgical course could be completed without complication. Blood loss was held at a minimum, and the patient could be extubated immediately postoperatively and remained in the intensive care unit for one night. The patient experienced no major complications and was discharged on the seventh postoperative day.

In the course of the follow-up 12 weeks after surgery, an upper gastrointestinal endoscopy was performed twice. Both showed a significant reduction in submucosal arterial collaterals. Endoscopic duplex ultrasound detected only a minimal residual arterial signal. The patient showed good quality of life after a 6 month follow-up and presented with no further gastrointestinal bleeding.

## Discussion and conclusion

Upper gastrointestinal bleeding are most commonly caused by either ulcers of various origin (50%) or gastric or oesophageal varices (20%). A large percentage of such duodenal and peptic ulcers are caused by helicobacter pylori induced gastroduodenitis, while only 20% are caused by nonsteroidal antiphlogistics [4,5] In this case, helicobacter pylori colonisation of the mucosa was not responsible for the development of an ulcer. Usually, the gastric bleeding due to ulcers is caused by erosion of the submucosal venous or arterial vessels. The most common location for such arterial bleeding is in the postpyloric region, due to erosion of the gastroduodenal artery. Though our patient presented with a small ulcer at the gastric fundus, it was unable to explain the cause of the varicose malformation.

Venous varices are often a symptom of portal hypertension, usually caused by liver cirrhosis [6,7]. In this case, venous blood flow is restricted through the portal vein and is redirected via venous collaterals to the right atrium. Such porto-caval collaterals are made noticeable through oesophageal and gastric varicosis, caput medusae, as well as rectal varicosis. Patients with gastric varicosis due to portal hypertension and ulcers bear a high risk of bleeding [8]. Arterial collaterals, as seen in this case, are generally not due to portal hypertension. Further excluding such a possible origin in our patient, a clinically relevant portal hypertension was ruled out using fibroscan, abdominal ultrasound, and computed tomography.

Splenic artery collaterals with gastric funcal varices as well as their possible complications have, until now, rarely been described [9-11]. The full pathomechanism remains unclear but thrombosis of the splenic artery can cause arterial collaterals via the route described. In addition, submucosal collaterals have been reported in patients with a congenital absence of the splenic artery [12,13].

In contrast, coiling of the splenic artery is a common procedure employed in the treatment of hypersplenism, otherwise known as the 'splenic artery steal syndrome,' in patients before and after liver transplantation. This procedure has been performed in cases where perfusion of the transplant is reduced and liver function impaired, or in order to improve liver perfusion prior to operation. While post-interventional abscesses or splenic infarct have been described and require splenectomy, gastric arterial collaterals have not been reported up till now, which underscores the rare nature of the present case [14,15].

N-butyl-2-cyanoacrylate injection for the treatment of gastric bleeding causes thrombosis of the renal vein [16], splenic artery occlusion [17] as well as portal vein thrombosis [18]. Because the varicose collaterals were clearly visible in the first gastroscopy, where N-butyl-2-cyanoacrylate injection was performed, we can safely assume that N-butyl-2-cyanoacrylate injection was not responsible for the splenic artery thrombosis. Blood sampling and thrombophilia diagnostic showed only a slight increase in factor VIII (approximately 164% n: 70-140). Increased factor VIII levels have previously been related to an increased risk for venous thromboembolism, especially in patients with cirrhosis and cancer [19,20] and low shear conditions [21], not though, for thromboembolism within the arterial circulation.

**Figure 5:**
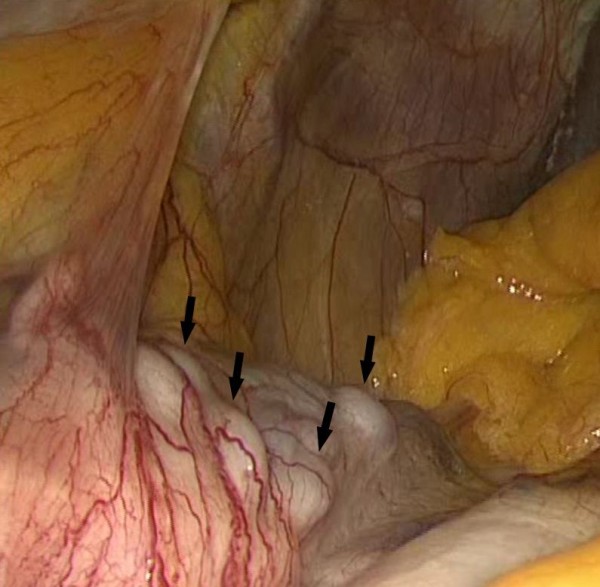
**Intraoperative situation showing the thick arterial collaterals along the gastric fundus**.

During the operation, massive collateral vessels via the short gastric arteries to the spleen were clearly visible (Figure 5). In addition, the cranial portion of the spleen showed signs of reduced perfusion. The collaterals via the short gastric arteries, as well as the main vessels of the spleen, were successfully ligated and cut during the laparoscopic procedure.

The cause for the thrombosis remains unclear, yet angiography has shown that the collaterals were sufficient to provide blood flow for the remaining spleen. Splenectomy was able to effectively reduce the collateral blood flow via the gastric arteries. This result was confirmed in the follow-up gastroscopy, which showed only remnants of the original varicosis. As such, we were able to conclude that thrombosis of the splenic artery was due to malformation of gastric submucosal arterial collaterals.

In the case of recurrent upper gastrointestinal bleedings from varicose-like structures, submucosal arterial collaterals must be excluded by endosonography via endoscopy. Should further ambiguity exist, further diagnostic, such as angiography and CT scan, are warranted.

If gastric submucous arterial collaterals can be shown, for example due to splenic artery thrombosis, laparoscopic splenectomy provides adequate treatment in preventing any recurrent bleeding.

The findings and procedures described in this paper were presented in video form at the 127th Congress of the German Society for Surgery on April 20-23, 2010 in Berlin, Germany.

## Consent

Written informed consent was obtained from the patient for publication of this case report and any accompanying images. A copy of the written consent is available for review by the Editor-in-Chief of this journal.

## Permissions

None of the material has been previously published.

## Competing interests

The authors declare that they have no competing interests.

## Authors' contributions

KT drafted and finalized the manuscript, prepared the figures, MB reviewed the manuscript and prepared the figures, FB prepared and provided radiologic figures and report, UN critically reviewed the manuscript and has given final approve for publication, HW and ST performed gastrointestinal endoscopy, duplex sonography of the stomach as well as the followup controls, MJ and KT performed the operation.

All authors read and approved the final manuscript.

## Pre-publication history

The pre-publication history for this paper can be accessed here:

http://www.biomedcentral.com/1471-2482/11/14/prepub
